# Real-world treatment trajectories of adults with newly diagnosed asthma or COPD

**DOI:** 10.1136/bmjresp-2023-002127

**Published:** 2024-02-27

**Authors:** Aniek F Markus, Peter R Rijnbeek, Jan A Kors, Edward Burn, Talita Duarte-Salles, Markus Haug, Chungsoo Kim, Raivo Kolde, Youngsoo Lee, Hae-Sim Park, Rae Woong Park, Daniel Prieto-Alhambra, Carlen Reyes, Jerry A Krishnan, Guy G Brusselle, Katia MC Verhamme

**Affiliations:** 1 Department of Medical Informatics, Erasmus University Medical Center, Rotterdam, The Netherlands; 2 Fundació Institut Universitari per a la Recerca a l'Atenció Primària de Salut Jordi Gol i Gurina (IDIAPJGol), Barcelona, Spain; 3 Centre for Statistics in Medicine (CSM), Nuffield Department of Orthopaedics, Rheumatology and Musculoskeletal Sciences (NDROMS), University of Oxford, Oxford, UK; 4 Institute of Computer Science, University of Tartu, Tartu, Estonia; 5 Department of Biomedical Sciences, Ajou University Graduate School of Medicine, Suwon, Republic of Korea; 6 Department of Allergy and Clinical Immunology, Ajou University School of Medicine, Suwon, Republic of Korea; 7 Breathe Chicago Center, Division of Pulmonary, Critical Care, Sleep, and Allergy, University of Illinois Chicago, Chicago, Illinois, USA; 8 Departments of Clinical Epidemiology and Respiratory Medicine, Erasmus University Medical Center, Rotterdam, The Netherlands; 9 Department of Respiratory Medicine, Ghent University Hospital, Ghent, Belgium; 10 Department of Infection Control & Epidemiology, OLV Hospital, Aalst, Belgium

**Keywords:** Asthma Pharmacology, COPD Pharmacology

## Abstract

**Background:**

There is a lack of knowledge on how patients with asthma or chronic obstructive pulmonary disease (COPD) are globally treated in the real world, especially with regard to the initial pharmacological treatment of newly diagnosed patients and the different treatment trajectories. This knowledge is important to monitor and improve clinical practice.

**Methods:**

This retrospective cohort study aims to characterise treatments using data from four claims (drug dispensing) and four electronic health record (EHR; drug prescriptions) databases across six countries and three continents, encompassing 1.3 million patients with asthma or COPD. We analysed treatment trajectories at drug class level from first diagnosis and visualised these in sunburst plots.

**Results:**

In four countries (USA, UK, Spain and the Netherlands), most adults with asthma initiate treatment with short-acting ß2 agonists monotherapy (20.8%–47.4% of first-line treatments). For COPD, the most frequent first-line treatment varies by country. The largest percentages of untreated patients (for asthma and COPD) were found in claims databases (14.5%–33.2% for asthma and 27.0%–52.2% for COPD) from the USA as compared with EHR databases (6.9%–15.2% for asthma and 4.4%–17.5% for COPD) from European countries. The treatment trajectories showed step-up as well as step-down in treatments.

**Conclusion:**

Real-world data from claims and EHRs indicate that first-line treatments of asthma and COPD vary widely across countries. We found evidence of a stepwise approach in the pharmacological treatment of asthma and COPD, suggesting that treatments may be tailored to patients’ needs.

**WHAT IS ALREADY KNOWN ON THIS TOPIC**

- There is a lack of global knowledge on the management of patients with asthma or chronic obstructive pulmonary disease (COPD) in the real world, especially with regard to the initial pharmacological treatment of newly diagnosed patients and the different treatment trajectories.

**WHAT THIS STUDY ADDS**

- With the help of innovative visualisations, we report substantial differences between databases and countries in the proportion of adults with newly diagnosed asthma or COPD who do not receive treatment and in the type of first treatment received.

**HOW THIS STUDY MIGHT AFFECT RESEARCH, PRACTICE OR POLICY**

- This first large-scale global characterisation study provides high-level insight into real-world treatment practices and helps to generate hypotheses for follow-up studies to address current gaps in clinical practice.

## Introduction

Asthma and chronic obstructive pulmonary disease (COPD) are prevalent chronic respiratory conditions with a large global health burden (21.6 and 74.4 million disability-adjusted life-years, respectively^1^). Both diseases have a negative impact on all aspects of life when not properly controlled and are responsible for (preventable) deaths, often as a result of acute exacerbations.^2^ Treatment is mainly organised via primary care and is aimed to minimise symptoms and prevent acute exacerbations. To support clinicians in the management of patients with asthma or COPD, several national and international guidelines have been developed which are frequently updated based on the latest research and insights.^3–9^ These guidelines suggest a stepwise treatment approach where treatment is initiated and tailored on the needs (ie, symptoms, severity, disease control and future risk) of the individual patient.

There is a lack of global knowledge on how patients with asthma or COPD are treated in the real world, especially with regard to the initial pharmacological treatment of newly diagnosed patients and the different treatment trajectories (encompassing both treatment step-up and treatment step-down strategies). Therefore, the purpose of this global characterisation study was to shed light on real-world treatment trajectories of newly diagnosed adults with asthma and COPD across different countries and continents. This descriptive study provides high-level insight into real-world treatment practices and helps to generate hypotheses for follow-up studies to address current gaps in clinical practice.

## Methods

### Study design

This is a retrospective cohort study based on routinely collected healthcare data, which has been mapped to the Observational Medical Outcomes Partnership Common Data Model (OMOP CDM).^10^ This mapping of data allows to conduct large-scale, multidatabase, international studies in an accurate, transparent and rapid manner.^11–13^

### Data sources

For this study, four claims databases and four electronic health record (EHR) databases from six countries were used: the USA, the UK, Spain, South Korea, the Netherlands and Estonia. Key characteristics of the databases used in this study are described in table 1, highlighting important differences between databases (see online supplemental file 1). All databases were mapped to the OMOP CDM.

10.1136/bmjresp-2023-002127.supp1 Supplementary data

**Table 1:** List of databases included in the study

Database name	Acronym	Country	Data type	Population size	Socioeconomic status	Drug prescription or dispensing available	Hospital treatments (inpatient visits)	Outpatient treatments
IBM MarketScan Commercial Claims and Encounters Database	CCAE	USA	Claims	160M	All	Dispensing	Yes	Yes
IBM MarketScan Multi-State Medicaid Database	Medicaid	USA	Claims	33M	Low	Dispensing	Yes	Yes
IBM MarketScan Medicare Supplemental Database	Medicare	USA	Claims	10M	All	Dispensing	Yes	Yes
Estonian Health Insurance Fund	EHIF	Estonia	Claims	1.4M	All	Dispensing	No	Yes
Clinical Practice Research Datalink	CPRD	UK	EHR, GP	15M	All	Prescription	No	Yes—primary care
Information System for Research in Primary Care	SIDIAP	Spain	EHR, GP	5.8M	All	Prescription	No	Yes—primary care
Integrated Primary Care Information	IPCI	The Netherlands	EHR, GP	2.5M	All	Prescription	No	Yes—primary care
Ajou University School of Medicine	AUSOM	South Korea	EHR, Hospital	3.3M	All	Prescription	Yes	Yes

### Study population

Within the databases, we identified two mutually exclusive cohorts: (1) a cohort of adults newly diagnosed with asthma (and no prior history of COPD) and (2) a cohort of adults newly diagnosed with COPD (and no prior history of asthma). For each cohort, we included patients available in the database with a first diagnosis from 1 January 2010 to 31 December 2019, having at least 1 year of database observation time prior to the first occurrence of a diagnosis record and at least 3 years of follow-up time since first diagnosis. This was required not only to have sufficient information on treatment history, but also to allow sufficient time following diagnosis to study treatment trajectories. Furthermore, we restricted the asthma cohort to patients aged 18 years or older and the COPD cohort to patients aged 40 years or older. Patients entered the cohort on the date of first diagnosis (ie, index date) and contributed to the follow-up time until they were transferred out of the database, death or the end of data collection, whichever occurred first. Asthma and COPD were defined by condition occurrence records based on SNOMED Clinical Terms (SNOMED CT) vocabulary, mapped from source diagnosis codes within each database (code list provided in online supplemental file 2).

### Respiratory drug classes

We studied treatment trajectories at drug class level and investigated the following types of respiratory drug classes: inhaled corticosteroids (ICS), short-acting ß2 agonists (SABA), long-acting ß2 agonists (LABA), short-acting muscarinic antagonists (SAMA), long-acting muscarinic antagonists (LAMA), leukotriene receptor antagonists (LTRA), xanthines, oral systemic glucocorticoids, phosphodiesterase-4 (PDE4), and biologics (including anti-IL4Rα, anti-IL5(R) and anti-IgE). Furthermore, we included four classes of fixed combinations of inhaled drugs: SABA-SAMA, LABA-LAMA, LABA-ICS and LABA-LAMA-ICS. With regard to systemic steroids, use of <30 days was considered a steroid burst whereas use of ≥30 days was considered maintenance therapy.^14 15^

Respiratory drug classes were defined based on RxNorm ingredient codes (a standard vocabulary used in the OMOP CDM), dose formulation of drugs recorded in the OMOP CDM, and where necessary the concept name of drugs (see online supplemental file 3). Missing records were interpreted as absence of treatments. Treatments were captured from the date of first diagnosis to the end of continuous database observation; there was no time window for the start of initial treatment.

### Baseline characteristics

To compare the study populations, we captured the patient characteristics of the asthma and COPD cohorts across databases. Covariates that were considered were age, sex, Charlson Comorbidity Index and specific comorbidities of interest (see online supplemental file 4 for details).

### Treatment trajectories

For all patients we investigated whether or not they received treatment during follow-up. For those who did, we studied the treatment trajectory, which is defined as the sequence of the respective respiratory drug classes over time. We first defined drug eras as continuous sequences of exposure records from the same class with a maximum gap of 30 days between exposures. Only drug eras of at least 5 days were included in the analysis. Switching was defined in case there was less than 30 days overlap with another drug class. If a patient received at least two drug classes at the same time for the full duration of one of the drug eras or with at least 30 days overlap, this was considered as combination therapy.

After constructing treatment trajectories for each patient, we counted the number of patients with the same treatment trajectory. Aggregated results (for trajectories that occur in at least 0.5% of the population) are presented in the form of sunburst plots. The sunburst plots show the sequence of treatments received over time but do not indicate the period of time between two consecutive treatments nor how long a patient receives a particular treatment. For a more detailed description of the constructed treatment trajectories, we refer to our earlier work.^16 17^ Study-specific settings are listed in online supplemental file 5.

### Stepwise treatment

Treatment switching and step-up/step-down treatment were also investigated. The type of switching between treatments was defined using two definitions: (1) a definition strictly following the Global Initiative for Asthma (GINA) guideline^3^ and Global Initiative for Chronic Obstructive Lung Disease (GOLD) guideline^7^ and (2) a broader definition capturing the clinical interpretation of the guidelines thereby categorising all possible switches (for full definitions see online supplemental file 6). The advantage of the first definition is that it is clean and in full accordance with clinical practice guideline recommendations; however, the second definition is more suited to match the heterogeneity of observational data and takes real-world circumstances into account. Guideline conformance was defined as the percentage of patients receiving follow-up treatment in accordance with the strict definition (ie, definition 1) after initial treatment, treatment step-up/step-down was analysed using the broader definition (ie, definition 2).

### Statistical methods

This study is characterising treatment trajectories and thus is descriptive in nature. No statistical comparisons were performed.

The R package needed to run the analysis on a database mapped to the OMOP CDM is available at: https://github.com/mi-erasmusmc/AsthmaCOPDTreatmentPatterns.

## Results

We present the main results in this section. All results can be explored in an interactive online Shiny application: https://mi-erasmusmc.shinyapps.io/AsthmaCOPDTreatmentPatterns/. The median follow-up time across databases was 5.6 years after first asthma or COPD diagnosis.

### Asthma

A total of 915 376 adults with asthma were identified. Demographic, socioeconomic and clinical characteristics at baseline differed substantially between databases. Table 2 shows that patients in Medicare were much older (mean age 73.0 vs 40.0–50.9 years for the other databases), patients in Medicaid were more often female (75.4% vs 61.4%–66.3%), and patients in Medicaid and Medicare had more comorbidities as indicated by a higher Charlson Comorbidity Index (1.4 and 2.4 vs 0.4–0.9 for the other databases). Coexisting conditions such as diabetes mellitus and hypertension were more prevalent in the US claims databases (CCAE, Medicaid and Medicare). Patients with asthma in the European primary care EHR databases (CPRD, IPCI and SIDIAP) were similar in terms of age and sex composition.

**Table 2:** Baseline characteristics of adults with asthma

Characteristic	CCAE (USA)	Medicaid (USA)	Medicare (USA)	EHIF (Estonia)	CPRD (UK)	SIDIAP (Spain)	IPCI (The Netherlands)	AUSOM (South Korea)
No of patients	572 637	127 803	48 544	22 949	44 983	85 088	10 793	2579
Sex: male, %	37.9	24.6	35.3	33.7	38.7	36.2	39.6	35.2
Age at index (years), mean	42.2	40	73	48.6	48.2	47.5	47.6	50.9
Charlson Comorbidity Index, mean	0.8	1.4	2.4	0.9	0.6	0.4	0.5	0.8
Common comorbid conditions, % (any time prior first diagnosis)								
Anxiety	18.1	35.9	12.4	9.5	22.8	24.2	20.4	4.1
Atopic disorders	3.1	2.3	2.7	3.5	11	3.1	12.8	7.2
Allergic rhinitis	34.1	26.6	25.4	14.9	17.7	23.7	24.7	25.8
Chronic rhinosinusitis	18.8	15.5	17	5.3	7.9	1.5	14.1	10.3
Depressive disorder	17.4	39	13.7	12.9	26	11.6	16.2	3.3
Diabetes mellitus	11.4	22	30.3	8	6.3	7.6	8.6	8.3
Gastro-oesophageal reflux disease	17.7	30.2	29.4	12	4	4	3	10.2
Nasal polyposis	1	0.4	1	1	1.8	1.4	0.5	1.6
Obesity	14.1	29.2	11.2	9.8	5.6	19.4	9.5	1.9
Lower respiratory tract infections (previous year)	25.4	27.5	34.6	36	3.9	23.6	15.7	16.5

**Figure 1:**
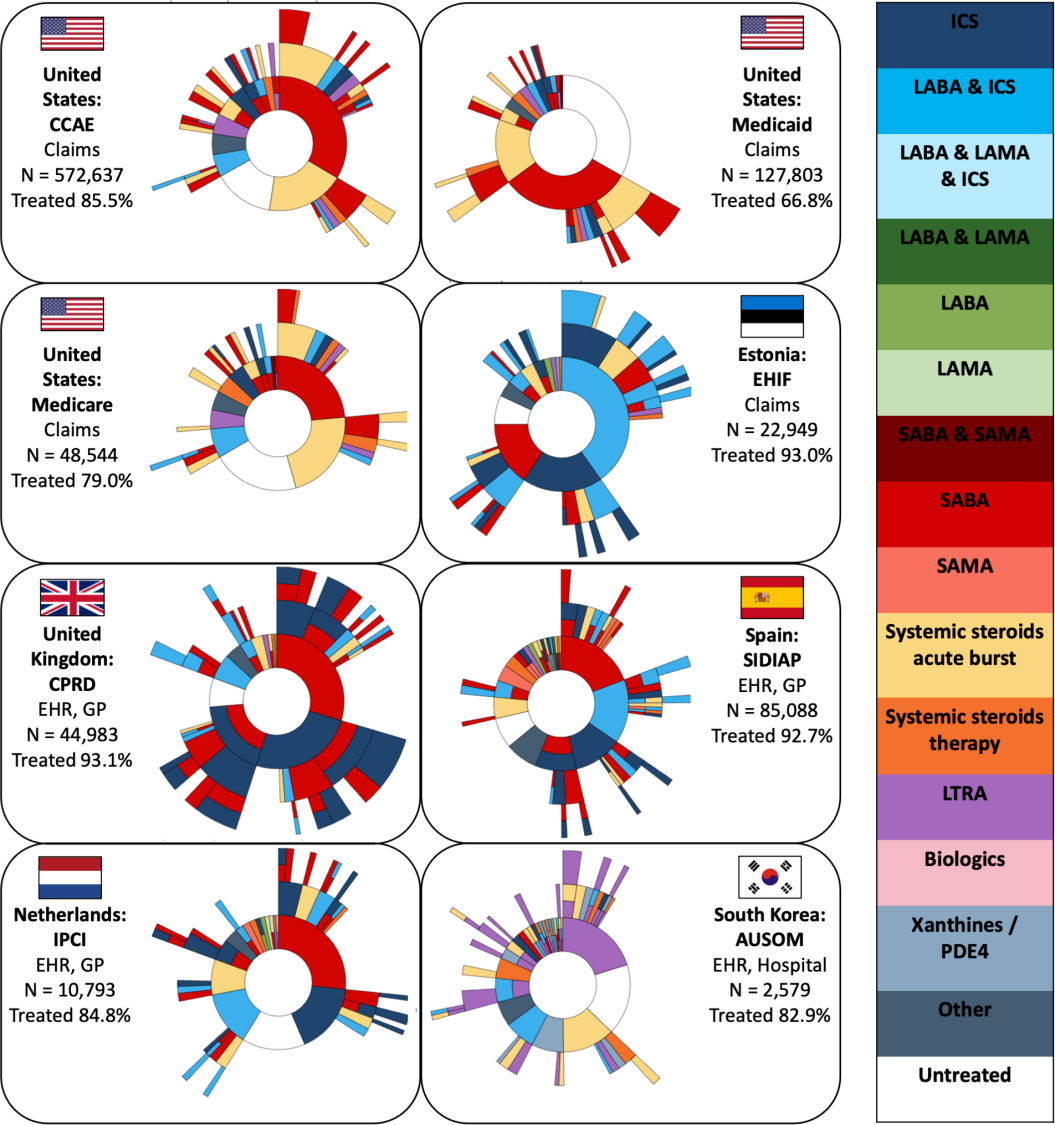
Sunburst plots of adults with asthma showing the first respiratory pharmacological treatment in the centre and subsequent pharmacological treatments in the surrounding outer layers. Each colour represents a respiratory drug class. A layer with multiple colours indicates a loose combination therapy. The number of patients (N) and percentage of patients treated are indicated for each database. EHR, electronic health record; GP, general practitioner; ICS, inhaled corticosteroids; LABA, long-acting ß2 agonists; LAMA, long-acting muscarinic antagonists; LTRA, leukotriene receptor antagonists; PDE4, phosphodiesterase-4; SABA, short-acting ß2 agonists; SAMA, short-acting muscarinic antagonists.

The sunburst plots for the treatment trajectories of newly diagnosed adults with asthma are presented in figure 1. The percentage of patients receiving any respiratory drug during follow-up time ranged from 66.8% in Medicaid (a claims database using drug dispensing data) to 93.1% in the CPRD database (an EHR database using drug prescription data). The most prevalent first-line treatment was SABA monotherapy in most databases (20.8%–47.4% of first-line treatments); exceptions to this are AUSOM (South Korea) and EHIF (Estonia). In AUSOM, the use of LTRA was the most common as first-line treatment and in EHIF a fixed combination of LABA-ICS. Other frequently used first-line treatments in adult patients with asthma were systemic steroid bursts (3.1%–27.9% across databases) and ICS monotherapy (2.3%–27.2% across databases). Second-line and higher-line treatments were common, but the type of respiratory drugs within these treatment lines varied widely between databases.

Next, we investigated what happened to patients following the end of first-line treatment. Across all databases, we found that 19.1%–38.4% of the patients did not receive subsequent treatment with a different drug class, 12.0%–23.9% proceeded to a higher treatment step and 6.6%–23.0% to a lower treatment step (see table 3). In most databases, the percentage of people increasing asthma therapy during follow-up was higher than the percentage of patients reducing their treatment (across databases on average 5.3% difference). Exceptions to this were AUSOM and EHIF, where more people stepped-down. The sensitivity analyses using the strict definition of treatment step-up/step-down showed similar patterns (see online supplemental file 7). The results for stepwise treatment showed that 2.6% (AUSOM) to 35.6% (CPRD) of follow-up treatments across databases was strictly conform to the GINA guidelines.

**Table 3:** Percentage of adults with asthma who switched, stepped-down or stepped-up respiratory pharmacological treatment after the first-line treatment (broad definition)

Label	CCAE (USA)	Medicaid (USA)	Medicare (USA)	EHIF (Estonia)	CPRD (UK)	SIDIAP (Spain)	IPCI (The Netherlands)	AUSOM (South Korea)
Step-up	13.1	11.9	12.0	20.6	23.9	21.0	17.6	14.3
Step-down	9.0	6.6	7.7	23.0	16.7	17.5	9.9	19.5
Switching	9.7	7.9	9.5	11.8	27.8	17.2	11.2	6.0
Start of acute exacerbation	20.1	20.9	18.3	10.6	6.5	12.4	12.1	14.3
End of acute exacerbation	15.9	14.9	15.9	3.3	4.6	8.4	8.1	14.9
No follow-up treatment*	32.0	37.5	36.0	29.9	19.1	20.6	38.4	30.6
Other	0.2	0.3	0.5	0.8	1.4	2.8	2.7	0.4

*Patients who did not receive medication of a different respiratory drug class after the first treatment, that is, patients who remained on the same treatment or who discontinued treatment.

### Chronic obstructive pulmonary disease

A total of 411 827 adults with COPD were identified across the databases. The baseline characteristics of adults with COPD are shown in table 4. Patients with COPD were typically older (54.9–75.9 for COPD vs 40.0–73.0 years for asthma) and had more coexisting conditions than adults with asthma, which was confirmed across databases by the substantially higher Charlson Comorbidity Index (1.8–4.1 vs 0.4–2.4 for asthma).

**Table 4:** Baseline characteristics of adults with chronic obstructive pulmonary disease

Characteristic	CCAE (USA)	Medicaid (USA)	Medicare (USA)	EHIF (Estonia)	CPRD (UK)	SIDIAP (Spain)	IPCI (The Netherlands)	AUSOM (South Korea)
No of patients	83 593	113 118	87 679	11 168	32 531	77 329	5365	1044
Sex: male, %	53.2	43.5	51.4	62.5	52.7	73	54.1	89.6
Age at index (years), mean	54.9	62.2	75.9	66.5	66	67.2	64.5	68.8
Charlson Comorbidity Index, mean	2.4	3.8	4.1	2.6	2.1	2.1	1.8	2.5
Common comorbid conditions, % (any time prior first diagnosis)								
Anxiety	21.4	36	12.6	5.8	24.7	19.6	18	1.8
Cerebrovascular disease	10.2	19.2	33.8	7.3	7.4	3.4	7.4	3.5
Depressive disorder	21.5	41.5	14.8	9.5	29.4	13.4	16.3	2.7
Diabetes mellitus	23	37	33.2	12.8	11.2	21.4	15.4	16.4
Heart failure	7.3	20.5	22.1	36.2	3.5	6.4	5.4	4.9
Hypertensive disorder	58.7	76.6	81.4	29.8	38.3	52.4	35.4	41.2
Ischaemic heart disease	14.2	20.2	28	23.9	15.4	10.5	16.2	16.1
Obesity	15.2	21.1	9	10.3	4.4	24.8	5.5	0.6
Lower respiratory tract infections (previous year)	30.4	32.6	30.8	39.1	5.3	27	22.4	20.6

**Figure 2:**
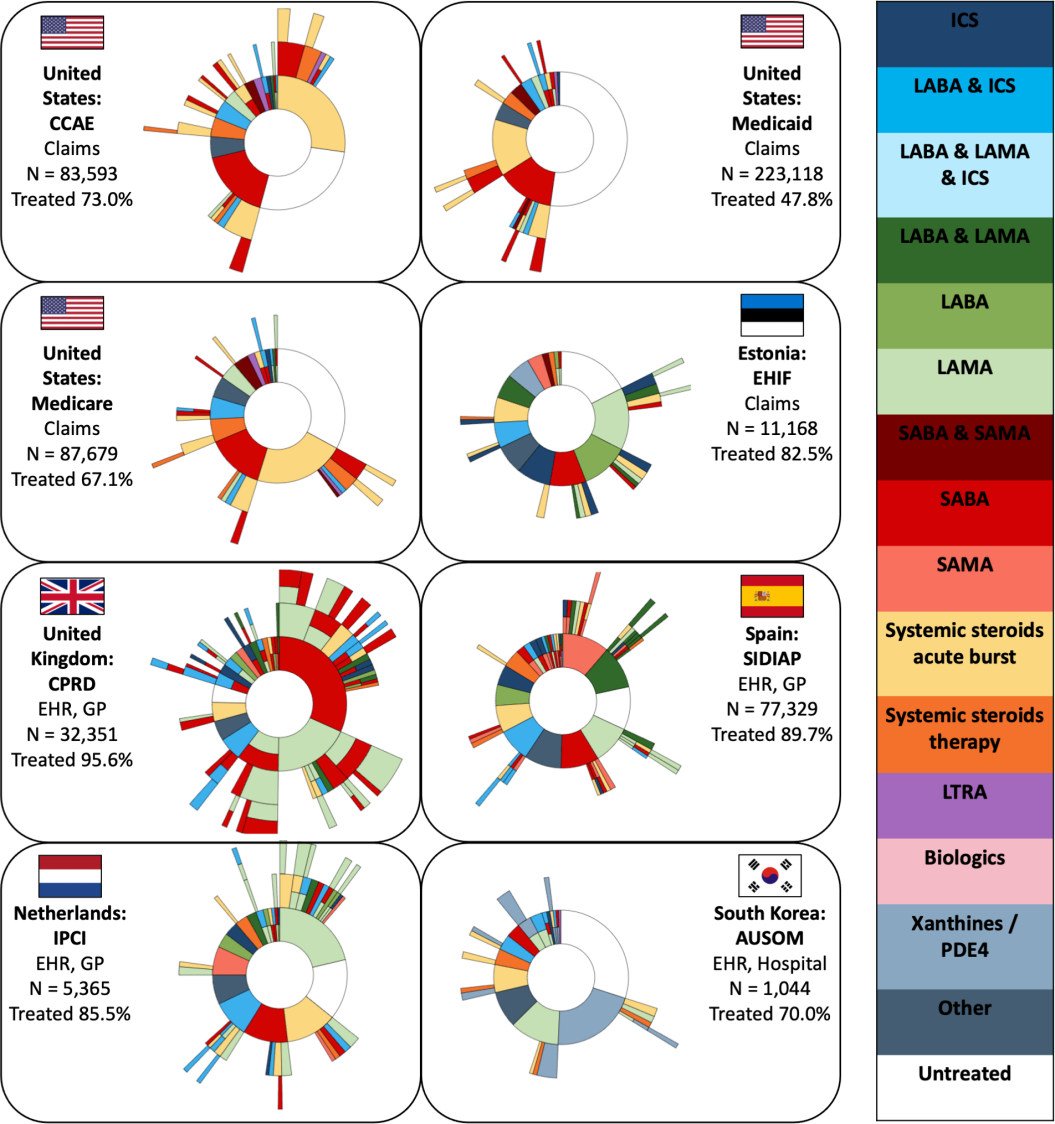
Sunburst plots of adults with chronic obstructive pulmonary disease showing the first respiratory pharmacological treatment in the centre and subsequent pharmacological treatments in the surrounding outer layers. Each colour represents a respiratory drug class. A layer with multiple colours indicates a loose combination therapy. The number of patients (N) and percentage of patients treated are indicated for each database. EHR, electronic health record; GP, general practitioner; ICS, inhaled corticosteroids; LABA, long-acting ß2 agonists; LAMA, long-acting muscarinic antagonists; LTRA, leukotriene receptor antagonists; PDE4, phosphodiesterase-4; SABA, short-acting ß2 agonists; SAMA, short-acting muscarinic antagonists.

The sunburst plots for the treatment trajectories of newly diagnosed adults with COPD are presented in figure 2. The percentage of patients receiving any respiratory drug during follow-up time ranged from 47.8% in Medicare to 95.6% in the CPRD database. The type of first-line treatment of adults with COPD varied across databases. In the USA, systemic steroid burst was the most common (28.6%–37.3% of first-line treatments), in the Netherlands (IPCI) and Estonia (EHIF) LAMA monotherapy (24.9% and 18.2%, respectively), in the UK (CPRD) SABA monotherapy (33.2%), in Spain (SIDIAP) SAMA monotherapy (12.6%) and in South Korea (AUSOM) xanthines monotherapy (29.5%). The type of second-line and third-line treatments in patients with COPD varied widely within and between databases as can be seen by the fragmented outer layers of the sunburst plots.

When exploring what happened to patients following the end of the first-line treatment period, we found that 11.6%–43.0% of patients with COPD did not receive subsequent treatment with a different drug class, 10.2%–32.9% stepped-up treatment and 6.6%–16.6% stepped-down treatment (see table 5). Within all databases, except AUSOM (South Korea), the percentage of adults with COPD who increased respiratory therapy was higher than the percentage reducing treatment (across databases on average 9.8% difference). This difference in percentage of patients who stepped-up and stepped-down within databases was on average larger for patients with COPD (9.8%) than patients with asthma (5.3%). Here as well, the strict definition showed similar results (see online supplemental file 7). The results for stepwise treatment showed that 6.3% (CCAE) to 28.6% (EHIF) of follow-up treatments across databases was strictly conform to the GOLD guidelines.

**Table 5:** Percentage of adults with chronic obstructive pulmonary disease who switched, stepped-down or stepped-up respiratory pharmacological treatment after the first-line treatment (broad definition)

Label	CCAE (USA)	Medicaid (USA)	Medicare (USA)	EHIF (Estonia)	CPRD (UK)	SIDIAP (Spain)	IPCI (The Netherlands)	AUSOM (South Korea)
Step-up	10.2	13.4	12.0	23.5	32.9	28.0	24.6	13.7
Step-down	6.6	7.8	7.5	13.7	13.3	14.6	12.1	16.6
Switching	8.6	11.3	9.4	9.2	25.4	17.0	10.8	9.6
Start of acute exacerbation	17.4	15.6	15.0	10.3	10.8	13.0	14.9	9.7
End of acute exacerbation	18.2	15.4	15.6	4.2	6.0	8.1	12.1	7.5
No follow-up treatment*	39.0	36.5	40.4	39.0	11.6	19.3	25.6	43.0
Other	0.04	0.01	0.01					

*Patients who did not receive medication of a different respiratory drug class after the first treatment, that is, patients who remained on the same treatment or who discontinued treatment.

## Discussion

We performed a large-scale characterisation of the pharmacological treatment of adults with obstructive airway diseases in eight large databases from six different countries to provide insight into global real-world treatment trajectories of patients with newly diagnosed asthma or COPD.

We found large differences in the proportion of patients with asthma or COPD receiving treatment across databases. Medicaid and Medicare (both US claims databases) had the largest percentage of untreated patients (33.2% and 21.0% for asthma; 52.2% and 32.9% for COPD), while European primary care EHR databases had the smallest percentage of patients not receiving any treatment (6.9%–15.2% for asthma; 4.4%–17.5% for COPD). With regard to first-line treatment, we found that in four countries (USA, UK, Spain and the Netherlands) most adults with asthma initiated treatment with SABA monotherapy (20.8%–47.4% of first-line treatments). Other frequent first-line treatments were (LABA-)ICS in Europe and systemic steroid bursts in the USA. In EHIF (Estonia), LABA-ICS and ICS monotherapy were frequently used first treatments, whereas in AUSOM (South Korea) LTRA monotherapy was the most common. With regard to COPD, LAMA monotherapy was the most frequent first-line treatment in IPCI (the Netherlands) and EHIF (Estonia), whereas in the UK (CPRD) and Spain (SIDIAP) this was monotherapy with a short-acting bronchodilator (SABA and SAMA, respectively). In the US databases, steroid bursts were frequently prescribed as first treatment. Systemic steroid bursts were rarely observed in combination with inhalation therapy for both asthma and COPD. Treatments for asthma COPD overlap (ACO) are different than the treatments observed for asthma or COPD alone (see online supplemental file 8). Patients with both diagnoses are more often treated and the variation in initial treatments is even larger.

The differences observed between the databases can be attributed to at least three main differences: (1) different types of data captured in the databases, (2) different patient populations enrolled in the databases and (3) differences between countries. First, claims databases use drug dispensing data, whereas EHR databases use drug prescription data. Since not all patients pick up their medication at the pharmacy (ie, primary non-adherence), EHR databases might overestimate the percentage of patients receiving treatment as compared with claims databases. As an example, the percentage of patients with COPD who do not receive treatment is higher in claims databases (in the USA and Estonia) as compared with EHR databases (in Europe and South Korea). Second, the impact of differences in patient populations (eg, demographic and socioeconomic characteristics) which are enrolled in the different databases is best exemplified among the claims databases in the USA. In CCAE, mainly wealthy younger subjects with a job and private insurance are enrolled, implicating less barriers to seek medical attention and receive medication. In contrast, older retired people in Medicare—showing the highest prevalence of comorbidities—and poorer people in Medicaid might have difficulties in consulting physicians and receiving preventive controller medications for asthma, leading to more frequent need of urgent care for acute exacerbations. Third, differences in treatments are also related to differences in healthcare systems between countries, availability and affordability of drugs, use of (inter)national guidelines, and sociocultural differences. Among the four EHR databases, for example, the high use of LTRA as first-line treatment in adults with asthma in South Korea contrasts with the infrequent use of LTRA in Europe.

The choice of first-line treatment was not always in line with recommendations by the (inter)national guidelines.^4^ Furthermore, we found that global guidelines conformance of follow-up treatments after initial treatment was low for both asthma (at most 35.6%) and COPD (at most 28.6%). Lack of adoption of international guidelines was also reported by other research groups in different regions.^18–20^ With regard to treatment switching and step-up/step-down treatment, we observed both increases and reductions in treatments for patients with asthma and COPD, suggesting that pharmacological treatments may be tailored to the needs (ie, symptoms, severity, disease control and future risk) of the individual patient. Note that even though guidelines have changed during the study period, observed treatment trajectories are stable across years within databases. Step-up/step-down of treatment has been advocated by guidelines during this entire period.

The observed differences in type of (first-line) treatments have also been observed by other research groups. High use of systemic steroids in adults with asthma in the USA was also reported by Tran *et al* who investigated data from CCAE/Medicaid/Medicare and reported that 65% of adults with asthma received treatment with oral corticosteroids.^21^ High use of LTRA in South Korea was also reported by Lee *et al* who investigated the prevalence of asthma (and its related mortality) in the National Health Insurance Sharing Service database of South Korea.^22^ In most databases, the most prevalent first-line treatment in asthma was SABA monotherapy, although GINA guidelines of 2014 already recommended use of low-dose ICS as controller therapy in mild asthma.^23^

We further observed that in patients with COPD, systemic steroids represented the majority of first-line treatments in the USA. Use of systemic steroids in the treatment of acute exacerbations is widely accepted meaning that, in the USA, it can be assumed that patients with COPD (as well as patients with asthma) present themselves for the first time with symptoms of an acute exacerbation.^6^ This delay in asthma and COPD diagnosis has been reported by other research groups, who attribute this delay either to a failure by physicians to recognise the disease or by patients to report their symptoms to their general practitioner.^24 25^ Furthermore, it is known that lower-income patients spend less on (costly) controller medications (eg, ICS and ICS-LABA), which causes rescue medications (ie, systemic steroids) to represent a larger proportion of the total drug use.^26^ In line with GOLD guidelines, in the other databases (except for AUSOM in South Korea) initial treatment of adults with COPD consisted of a bronchodilator, either short acting or long acting.^7^ The choice of bronchodilator depends on the number of previous COPD exacerbations and symptom control. Earlier work studying treatment for COPD in SIDIAP during the period 2007–2012 reported that the most frequently prescribed first-line treatments were short-acting bronchodilators (17.7%) and LABA-ICS (17.3%).^27^ In that study, use of LABA-ICS was somewhat higher to what we observed, which might in part be explained by the fact that they did not exclude patients with ACO. Use of xanthines in patients with COPD (as first-line treatment) was still high in South Korea whereas this is no longer recommended according to GOLD guidelines. This finding was confirmed in a cohort of patients with mild-to-moderate COPD selected from the Korean National Health and Nutrition Examination Survey data between 2007 and 2012,^28^ reporting high use of methylxanthines (68%) compared with inhalation therapy (37%). Widespread use of oral methylxanthines despite guideline recommendations was related to the accessibility of these drugs and the fact that these oral drugs are easy to administer.^28^

This is the first, large, global characterisation study of real-world treatment trajectories of adults with obstructive airway diseases. The strengths of this study include the diversity and size of included databases, the transparency and reproducibility of the analysis because of the publicly available study package, and the novelty of the analysis and visualisations. However, as for all observational studies, our study has limitations too. First, the study cohorts are based on the presence of SNOMED CT and RxNorm codes, which might lead to misclassification in the case of suboptimal coding. Because of the sample size, it was not possible to manually validate patients and thus we cannot quantify the value of the potential misclassification. Second, patients with ‘newly diagnosed’ asthma might have had asthma during childhood; however, the minimum database observation time of 1 year prior to inclusion and the use of all available medical history allowed us to check for prevalent asthma prior to study start. Third, there might be differences in the availability of information between databases (eg, claims vs EHR and primary care vs hospital). Finally, it should be noted that the analysis of treatment trajectories in observational data is limited to information on drug prescription/dispensing, whereas we do not have information on treatment adherence. Hence, it is not possible to infer actual treatment, which might lead to an overestimation of drug use.

To improve clinical practice, it is important to study differences between (inter)national guidelines and actual drug use in real-world settings to better understand the (lack of) adoption of guidelines. Further research is necessary to study changes in treatment trajectories over time (eg, in response to novel recommendations of guidelines) and to investigate the relation between different treatment trajectories and clinical outcomes (such as acute exacerbations, emergency department visits, hospital admissions and mortality).

## Data Availability

Data may be obtained from a third party and are not publicly available.
