# Supplementary material for: Real-world treatment trajectories of adults with newly diagnosed asthma or COPD
Source: BMJ Open Respir Res. 2024 Feb 27;11(1):e002127. doi: 10.1136/bmjresp-2023-002127 (PMC10900306; doi:10.1136/bmjresp-2023-002127)
Supplement: Supplementary data [file bmjresp-2023-002127supp001.pdf]

ONLINE SUPPLEMENT

Title

Real-world treatment trajectories of adults with newly diagnosed asthma or COPD

Authors

Aniek F. Markus[1†] MSc,

Peter R. Rijnbeek[1] PhD,

Jan A. Kors[1] PhD,

Edward Burn[2,3] DPhil,

Talita Duarte-Salles[3] MPH, PhD,

Markus Haug[4],

Chungsoo Kim[5] PharmD,

Raivo Kolde[4], PhD,

Youngsoo Lee[6], MD,

Hae-Sim Park[6], MD, PhD,

Rae Woong Park[5] MD, PhD,

Daniel Prieto-Alhambra[1,2] MD, PhD,

Carlen Reyes[3], MD

Jerry A. Krishnan[7], MD, PhD

Guy G. Brusselle[8,9] MD, PhD,*

Katia M.C. Verhamme[1,10] MD, PhD*

*Both authors contributed equally to the supervision of the research work and manuscript.

TABLE OF CONTENTS

ONLINE SUPPLEMENT 1 – ADDITIONAL DATABASE INFORMATION 3

ONLINE SUPPLEMENT 2 – STUDY POPULATIONS 7

ONLINE SUPPLEMENT 3 – RESPIRATORY DRUG CLASSES 18

ONLINE SUPPLEMENT 4 – BASELINE CHARACTERISTICS 25

ONLINE SUPPLEMENT 5 – STUDY SETTINGS TREATMENT TRAJECTORIES 26

ONLINE SUPPLEMENT 6 – STEP-WISE TREATMENT 27

ONLINE SUPPLEMENT 7 – RESULTS TREATMENT STEP-UP/STEP-DOWN 36

ONLINE SUPPLEMENT 8 – SENSITIVITY ANALYSIS 38

ONLINE SUPPLEMENT 9 – POOLED RESULTS 43

REFERENCES 44

Online Supplement 1 – Additional database information

List of descriptions:

- IBM MarketScan Commercial Claims and Encounters (CCAE), United States

This database contains data from individuals enrolled in United States employer-sponsored insurance health plans, for example, employees, their spouses, and dependents. The data include inpatient and outpatient medical claims, diagnosis codes, and outpatient pharmacy dispensing claims. The patients in CCAE are between 0 and 65 years of age. Additionally, it captures laboratory test results for a subset of the covered population. This administrative claims database includes a variety of fee-for-service, preferred provider organizations, and capitated health plans. The database covers the period January 2000 until May 2021.

- IBM MarketScan Multi-State Medicaid (Medicaid), United States

This database contains adjudicated United States health insurance claims for Medicaid (government aided insurance) enrollees from multiple states. It includes hospital discharge diagnoses, outpatient diagnoses and procedures, outpatient pharmacy dispensing claims, as well as ethnicity and Medicare eligibility for enrollees, mostly, under 65 years of age. Members maintain their same identifier even if they leave the system for a brief period however the dataset lacks lab data. The database covers the period January 2006 until January 2021.

- IBM MarketScan Medicare Supplemental (Medicare), United States

This database represents health services of retirees in the United States with primary or Medicare supplemental coverage through privately insured fee-for-service, point-of-service, or capitated health plans. These data include adjudicated health insurance claims (e.g.

inpatient, outpatient, and outpatient pharmacy). Additionally, it captures laboratory tests for a subset of the covered lives. The database covers the period January 2000 until May 2021.

- Estonian Health Insurance Fund (EHIF), Estonia

This database contains claims in national insurer Estonian Health Insurance Fund and its digital prescription service that handles more than 99% of the prescriptions in Estonia. The dataset is obtained according to the University of Tartu ethics committee approval (299/T-21). As the data comes from a national insurer, it represents all Estonian patients at all levels of healthcare. For each patient, there are all the recorded ICD-10 diagnosis codes from the insurance claims and all prescriptions showing ATC codes for active ingredient together with prescription and dispensation times. The database covers the period January 2011 until December 2019.

- Clinical Practice Research Datalink (CPRD), United Kingdom

This database is a governmental, not-for-profit research service, jointly funded by the NHS National Institute for Health Research (NIHR) and the Medicines and Healthcare products Regulatory Agency (MHRA), a part of the Department of Health, United Kingdom (UK). CPRD consists of data collected from UK primary care for all ages. This includes conditions, observations, measurements, and procedures that the general practitioner is made aware of in additional to any prescriptions as prescribed by the general practitioner. In addition to primary care, there are also linked secondary care records for a small number of people. The major data elements contained within this database are outpatient prescriptions given by the general practitioner (coded with Multilex codes) and outpatient clinical, referral,

immunization or test events that the general practitioner knows about (coded in Read or ICD10 or LOINC codes). The database also contains the patients' year of births and any date of deaths. The database covers the period January 1995 until July 2020.

- Supplemental Database Information System for Research in Primary Care (SIDIAP), Spain

This primary care records database covers approximately 80% of the population of Catalonia, North-East Spain, with data from 328 primary care centres. SIDIAP was linked to the minimum basic set of hospital discharge data (CMBD-HA), which includes diagnosis and procedures registered during hospital admissions. The database contains information on health conditions as captured via diagnoses registered by healthcare professionals, along with prescriptions made. The database covers the period January 2006 until May 2021.

- Integrated Primary Care Information (IPCI), Netherlands

This database contains longitudinal data of patients of a group of general practitioners (GPs) in The Netherlands. Started in 1992 by the Department of Medical Informatics of the Erasmus University Medical Center, Rotterdam, to enable better post-marketing surveillance of drugs, the IPCI database is used in a large number of studies including drug use, disease prevalence, drug safety and effectiveness studies and methodological research. It consists of 2.5 million patient records, with median follow-up of 4.8 years, and includes patient demographics, information about contacts with GPs, symptoms, diagnoses, laboratory and clinical measurements, prescriptions, and information on use of secondary care. Drugs not prescribed in the GP setting might be underreported. Indications are available as diagnoses by the GPs and, indirectly, from secondary care providers but the

latter might not be complete. The database covers the period January 2006 until August 2020.

- Ajou University School of Medicine (AUSOM), South Korea

This database is a single electronic health record database of Ajou University Medical Center in South Korea. Ajou University Medical Center is a tertiary teaching hospital in South Korea, therefore, most patients are needed professional care from medical specialists. Most patients have to visit this tertiary center with a medical request form written from the primary or secondary institutions according to the healthcare system of South Korea. The AUSOM data includes diagnoses, medications, lab tests, surgeries and procedures, observations, and visit records. All prescribing includes outpatient and inpatient prescriptions. The database covers the period January 1994 until June 2020.

Table E1: Strengths and limitations of data types included in this study

Data type	Drug data	Strengths	Limitations/biases
EHR, GP	Drug prescriptions	Information on prescribing patterns	Overestimation of actual treatment due to primary non-adherence and lack of information on treatment intake
EHR, Hospital	Drug prescriptions	Information on prescribing patterns	Overestimation of actual treatment due to primary non-adherence and lack of information on treatment intake
Claims	Drug dispensing	Information on filled prescriptions	Overestimation of actual treatment due to lack of information on treatment intake

Markus AF, *et al. BMJ Open Respir Res* 2024; 11:e002127. doi: 10.1136/bmjresp-2023-002127

<u>Online Supplement 2 – Study populations</u>

i) Asthma

Initial Event Cohort:

People with continuous observation of 365 days before and 1,095 days after event may enter the cohort when observing any of the following:

- Condition occurrence of Asthma* for the first time in the person's history.

Inclusion Criteria:

- Age ≥ 18 years AND,
- No condition occurrences of COPD** in their history or during follow-up time AND,
- Index date after December 31, 2009 and before December 31, 2019.

Cohort Exit: the person exits the cohort at the end of continuous observation.

ii) COPD

Initial Event Cohort:

People with continuous observation of 365 days before and 1,095 days after event may enter the cohort when observing any of the following:

- Condition occurrence of COPD** for the first time in the person's history.

Inclusion Criteria:

- Age ≥ 40 years AND,
- No condition occurrences of Asthma* in their history or during follow-up time AND,
- Index date after December 31, 2009 and before December 31, 2019.

Markus AF, *et al. BMJ Open Respir Res* 2024; 11:e002127. doi: 10.1136/bmjresp-2023-002127

Cohort Exit: the person exits the cohort at the end of continuous observation.

Table E2: Number of patients per year with asthma.

Characteristic	CCAE (United States)	Medicaid (United States)	Medicare (United States)	EHIF (Estonia)	CPRD (United Kingdom)	SIDIAP (Spain)	IPCI (Nether lands)	AUSOM (South Korea)
Year 2010	75770	7976	8593		7891	11360	929	362
Year 2011	79930	7540	7012		7062	9502	908	413
Year 2012	59507	7500	5917	6357	7023	10100	1250	301
Year 2013	62579	13574	6313	4735	6123	11266	1474	253
Year 2014	74769	12232	6883	4307	5578	10620	1545	252
Year 2015	69007	25589	4500	3901	4751	9822	1705	231
Year 2016	63575	28477	4276	3649	4576	8312	1941	354
Year 2017	63471	24869	3616		1979	7973	1041	258
Year 2018+	24029	46	1434			6133		155
**Total**	**572,637**	**127,803**	**48,544**	**22,949**	**44,983**	**85,088**	**10,793**	**2,579**

Table E3: Number of patients per year with COPD.

Characteristic	CCAE (United States)	Medicaid (United States)	Medicare (United States)	EHIF (Estonia)	CPRD (United Kingdom)	SIDIAP (Spain)	IPCI (Nether lands)	AUSOM (South Korea)
Year 2010	10914	8325	15696		5334	8313	509	105
Year 2011	11582	8094	13723		5351	7740	540	163
Year 2012	9153	8031	13517	2609	4976	9671	642	129
Year 2013	8802	14242	13019	2242	4177	11023	747	116
Year 2014	10507	11815	12324	2136	3726	10386	749	110
Year 2015	10343	18658	6761	2163	3745	9534	832	109
Year 2016	10027	22956	6596	2018	3655	8230	904	139
Year 2017	8966	20920	4623		1567	7365	442	98
Year 2018+	3299	77	1420			5067		75
**Total**	**83,593**	**113,118**	**87,679**	**11,168**	**32,531**	**77,329**	**5,365**	**1,044**

Table E4: Code list for asthma*.

Concept ID	Name	Class	Domain ID	Vocabulary
252658	Intrinsic asthma without status asthmaticus	Clinical Finding	Condition	SNOMED
257581	Exacerbation of asthma	Clinical Finding	Condition	SNOMED
312950	IgE-mediated allergic asthma	Clinical Finding	Condition	SNOMED
313236	Cough variant asthma	Clinical Finding	Condition	SNOMED
317009	Asthma	Clinical Finding	Condition	SNOMED
443801	Exercise-induced asthma	Clinical Finding	Condition	SNOMED
761844	Inhaled steroid-dependent asthma	Clinical Finding	Condition	SNOMED
764677	Persistent asthma	Clinical Finding	Condition	SNOMED
764949	Persistent asthma, well controlled	Clinical Finding	Condition	SNOMED
4015819	Asthma disturbs sleep weekly	Clinical Finding	Condition	SNOMED
4015947	Asthma causing night waking	Clinical Finding	Condition	SNOMED
4017025	Asthma disturbing sleep	Clinical Finding	Condition	SNOMED
4017026	Asthma not limiting activities	Clinical Finding	Condition	SNOMED
4017182	Asthma disturbs sleep frequently	Clinical Finding	Condition	SNOMED
4017183	Asthma not disturbing sleep	Clinical Finding	Condition	SNOMED
4017184	Asthma never disturbs sleep	Clinical Finding	Condition	SNOMED
4017293	Asthma never restricts exercise	Clinical Finding	Condition	SNOMED
4022592	Millers’ asthma	Clinical Finding	Condition	SNOMED
4051466	Childhood asthma	Clinical Finding	Condition	SNOMED
4075237	Brittle asthma	Clinical Finding	Condition	SNOMED
4110051	Mixed asthma	Clinical Finding	Condition	SNOMED
4119298	Late onset asthma	Clinical Finding	Condition	SNOMED
4120261	Sulfite-induced asthma	Clinical Finding	Condition	SNOMED
4123253	Hay fever with asthma	Clinical Finding	Condition	SNOMED
4125022	Acute asthma	Clinical Finding	Condition	SNOMED

Concept ID	Name	Class	Domain ID	Vocabulary
4138760	Exacerbation of intermittent asthma	Clinical Finding	Condition	SNOMED
4141978	Intermittent asthma	Clinical Finding	Condition	SNOMED
4142738	Moderate persistent asthma	Clinical Finding	Condition	SNOMED
4143474	Bakers' asthma	Clinical Finding	Condition	SNOMED
4143828	Mild persistent asthma	Clinical Finding	Condition	SNOMED
4145356	Severe persistent asthma	Clinical Finding	Condition	SNOMED
4145497	Intrinsic asthma	Clinical Finding	Condition	SNOMED
4146581	Mild intermittent asthma	Clinical Finding	Condition	SNOMED
4152292	Asthma causes night symptoms 1 to 2 times per month	Clinical Finding	Condition	SNOMED
4152418	Asthma never causes daytime symptoms	Clinical Finding	Condition	SNOMED
4152420	Asthma treatment compliance unsatisfactory	Clinical Finding	Condition	SNOMED
4152911	Asthma causes daytime symptoms most days	Clinical Finding	Condition	SNOMED
4152913	Severe asthma	Clinical Finding	Condition	SNOMED
4155468	Mild asthma	Clinical Finding	Condition	SNOMED
4155469	Moderate asthma	Clinical Finding	Condition	SNOMED
4155470	Occasional asthma	Clinical Finding	Condition	SNOMED
4155473	Asthma treatment compliance satisfactory	Clinical Finding	Condition	SNOMED
4156136	Asthma causes daytime symptoms 1 to 2 times per month	Clinical Finding	Condition	SNOMED
4161595	Asthma daytime symptoms	Clinical Finding	Condition	SNOMED
4191479	Allergic asthma	Clinical Finding	Condition	SNOMED
4191827	Asthma night-time symptoms	Clinical Finding	Condition	SNOMED
4194289	Asthma – currently active	Clinical Finding	Condition	SNOMED

Markus AF, et al. BMJ Open Respir Res 2024; 11:e002127. doi: 10.1136/bmjresp-2023-002127

Concept ID	Name	Class	Domain ID	Vocabulary
4206340	Asthma without status asthmaticus	Clinical Finding	Condition	SNOMED
4211530	Wood asthma	Clinical Finding	Condition	SNOMED
4212099	Occupational asthma	Clinical Finding	Condition	SNOMED
4217558	Detergent asthma	Clinical Finding	Condition	SNOMED
4225553	Cheese-makers’ asthma	Clinical Finding	Condition	SNOMED
4225554	Isocyanate induced asthma	Clinical Finding	Condition	SNOMED
4232595	Platinum asthma	Clinical Finding	Condition	SNOMED
4245676	Chemical-induced asthma	Clinical Finding	Condition	SNOMED
4271333	Extrinsic asthma without status asthmaticus	Clinical Finding	Condition	SNOMED
4279553	Eosinophilic asthma	Clinical Finding	Condition	SNOMED
4301938	Tea-makers’ asthma	Clinical Finding	Condition	SNOMED
4309833	Non-IgE mediated allergic asthma	Clinical Finding	Condition	SNOMED
4312524	Substance induced asthma	Clinical Finding	Condition	SNOMED
35609846	Life threatening acute exacerbation of extrinsic asthma	Clinical Finding	Condition	SNOMED
35609847	Life threatening acute exacerbation of non-allergic asthma	Clinical Finding	Condition	SNOMED
36674599	Asthma never causes night symptoms	Clinical Finding	Condition	SNOMED
36684328	Acute severe exacerbation of allergic asthma	Clinical Finding	Condition	SNOMED
36684335	Exacerbation of allergic asthma due to infection	Clinical Finding	Condition	SNOMED
37108580	Severe controlled persistent asthma	Clinical Finding	Condition	SNOMED
37108581	Severe uncontrolled persistent asthma	Clinical Finding	Condition	SNOMED
37109103	Oral steroid-dependent asthma	Clinical Finding	Condition	SNOMED

Concept ID	Name	Class	Domain ID	Vocabulary
37116845	Acute severe refractory exacerbation of asthma	Clinical Finding	Condition	SNOMED
40481763	Acute exacerbation of chronic asthmatic bronchitis	Clinical Finding	Condition	SNOMED
40483397	Seasonal asthma	Clinical Finding	Condition	SNOMED
42535716	Asthma in pregnancy	Clinical Finding	Condition	SNOMED
42536207	Life threatening acute exacerbation of asthma	Clinical Finding	Condition	SNOMED
42536208	Moderate acute exacerbation of asthma	Clinical Finding	Condition	SNOMED
42536649	Uncomplicated non-allergic asthma	Clinical Finding	Condition	SNOMED
42538744	Exacerbation of allergic asthma	Clinical Finding	Condition	SNOMED
42539549	Uncomplicated allergic asthma	Clinical Finding	Condition	SNOMED
43530745	Asthma with irreversible airway obstruction	Clinical Finding	Condition	SNOMED
44805087	Asthma causes night time symptoms 1 to 2 times per week	Clinical Finding	Condition	SNOMED
44805089	Asthma causes symptoms most nights	Clinical Finding	Condition	SNOMED
44810117	Chronic asthma with fixed airflow obstruction	Clinical Finding	Condition	SNOMED
45766727	Allergic asthma due to Dermatophagoides pteronyssinus	Clinical Finding	Condition	SNOMED
45766728	Allergic asthma due to Dermatophagoides farinae	Clinical Finding	Condition	SNOMED
45768910	Uncomplicated asthma	Clinical Finding	Condition	SNOMED
45768911	Exacerbation of mild persistent asthma	Clinical Finding	Condition	SNOMED
45768912	Exacerbation of severe persistent asthma	Clinical Finding	Condition	SNOMED

Markus AF, *et al. BMJ Open Respir Res* 2024; 11:e002127. doi: 10.1136/bmjresp-2023-002127

Concept ID	Name	Class	Domain ID	Vocabulary
45768963	Uncomplicated mild persistent asthma	Clinical Finding	Condition	SNOMED
45768964	Uncomplicated moderate persistent asthma	Clinical Finding	Condition	SNOMED
45768965	Uncomplicated severe persistent asthma	Clinical Finding	Condition	SNOMED
45769350	Acute severe exacerbation of severe persistent asthma	Clinical Finding	Condition	SNOMED
45769351	Acute severe exacerbation of moderate persistent asthma	Clinical Finding	Condition	SNOMED
45769352	Acute severe exacerbation of mild persistent asthma	Clinical Finding	Condition	SNOMED
45769438	Acute severe exacerbation of asthma	Clinical Finding	Condition	SNOMED
45769441	Acute exacerbation of allergic asthma	Clinical Finding	Condition	SNOMED
45769442	Acute severe exacerbation of allergic asthma	Clinical Finding	Condition	SNOMED
45769443	Acute severe exacerbation of intrinsic asthma	Clinical Finding	Condition	SNOMED
45771045	Acute exacerbation of asthma	Clinical Finding	Condition	SNOMED
45772937	Exacerbation of moderate persistent asthma	Clinical Finding	Condition	SNOMED
45773005	Acute exacerbation of intrinsic asthma	Clinical Finding	Condition	SNOMED
46269767	Acute severe exacerbation of asthma co-occurrent with allergic rhinitis	Clinical Finding	Condition	SNOMED
46269770	Severe persistent allergic asthma	Clinical Finding	Condition	SNOMED
46269771	Acute severe exacerbation of severe persistent asthma co-occurrent with allergic rhinitis	Clinical Finding	Condition	SNOMED

Concept ID	Name	Class	Domain ID	Vocabulary
46269772	Severe persistent allergic asthma controlled	Clinical Finding	Condition	SNOMED
46269773	Severe persistent asthma controlled co-occurrent with allergic rhinitis	Clinical Finding	Condition	SNOMED
46269774	Severe persistent allergic asthma uncontrolled	Clinical Finding	Condition	SNOMED
46269775	Severe persistent asthma uncontrolled co-occurrent with allergic rhinitis	Clinical Finding	Condition	SNOMED
46269776	Mild persistent allergic asthma	Clinical Finding	Condition	SNOMED
46269777	Acute severe exacerbation of mild persistent allergic asthma co-occurrent with allergic rhinitis	Clinical Finding	Condition	SNOMED
46269778	Mild persistent asthma controlled	Clinical Finding	Condition	SNOMED
46269779	Mild persistent asthma controlled co-occurrent with allergic rhinitis	Clinical Finding	Condition	SNOMED
46269780	Mild persistent allergic asthma uncontrolled	Clinical Finding	Condition	SNOMED
46269781	Mild persistent asthma uncontrolled	Clinical Finding	Condition	SNOMED
46269782	Mild persistent asthma uncontrolled co-occurrent with allergic rhinitis	Clinical Finding	Condition	SNOMED
46269783	Moderate persistent asthma controlled	Clinical Finding	Condition	SNOMED
46269784	Moderate persistent allergic asthma	Clinical Finding	Condition	SNOMED
46269785	Acute severe exacerbation of moderate persistent asthma co-occurrent with allergic rhinitis	Clinical Finding	Condition	SNOMED
46269786	Moderate persistent allergic asthma controlled	Clinical Finding	Condition	SNOMED

Concept ID	Name	Class	Domain ID	Vocabulary
46269787	Moderate persistent controlled asthma co-occurrent with allergic rhinitis	Clinical Finding	Condition	SNOMED
46269788	Moderate persistent allergic asthma uncontrolled	Clinical Finding	Condition	SNOMED
46269789	Moderate persistent asthma uncontrolled co-occurrent with allergic rhinitis	Clinical Finding	Condition	SNOMED
46269790	Moderate persistent asthma uncontrolled	Clinical Finding	Condition	SNOMED
46269802	Chronic obstructive asthma co-occurrent with acute exacerbation of asthma	Clinical Finding	Condition	SNOMED
46270028	Severe persistent asthma co-occurrent with allergic rhinitis	Clinical Finding	Condition	SNOMED
46270029	Mild persistent asthma co-occurrent with allergic rhinitis	Clinical Finding	Condition	SNOMED
46270030	Intermittent asthma co-occurrent with allergic rhinitis	Clinical Finding	Condition	SNOMED
46270082	Acute exacerbation of mild persistent asthma	Clinical Finding	Condition	SNOMED
46270322	Intermittent asthma uncontrolled	Clinical Finding	Condition	SNOMED
46270573	Intermittent asthma well controlled	Clinical Finding	Condition	SNOMED
46273452	Acute exacerbation of asthma co-occurrent with allergic rhinitis	Clinical Finding	Condition	SNOMED
46273454	Moderate persistent asthma co-occurrent with allergic rhinitis	Clinical Finding	Condition	SNOMED
46273462	Acute severe exacerbation of moderate persistent allergic asthma	Clinical Finding	Condition	SNOMED

Markus AF, et al. BMJ Open Respir Res 2024; 11:e002127. doi: 10.1136/bmjresp-2023-002127

Concept ID	Name	Class	Domain ID	Vocabulary
46273487	Acute exacerbation of moderate persistent asthma	Clinical Finding	Condition	SNOMED
46273635	Steroid dependent asthma	Clinical Finding	Condition	SNOMED
46274059	Acute severe exacerbation of severe persistent allergic asthma	Clinical Finding	Condition	SNOMED
46274060	Mild persistent allergic asthma controlled	Clinical Finding	Condition	SNOMED
46274124	Acute severe exacerbation of mild persistent allergic asthma	Clinical Finding	Condition	SNOMED
46287068	At risk of severe asthma exacerbation	Clinical Finding	Condition	SNOMED
4057952	Meat-wrappers’ asthma	Clinical Finding	Condition	SNOMED
4080516	Printers’ asthma	Clinical Finding	Condition	SNOMED
4119300	Colophony asthma	Clinical Finding	Condition	SNOMED

Table E5: Code list for COPD**.

Concept ID	Name	Class	Domain ID	Vocabulary
255573	Chronic obstructive lung disease	Clinical Finding	Condition	SNOMED
257004	Acute exacerbation of chronic obstructive airways disease	Clinical Finding	Condition	SNOMED
36685451	GOLD (Global Initiative for Chronic Obstructive Lung Disease) 2017 group A	Clinical Finding	Condition	SNOMED
36685452	GOLD (Global Initiative for Chronic Obstructive Lung Disease) 2017 group B	Clinical Finding	Condition	SNOMED
36685453	GOLD (Global Initiative for Chronic Obstructive Lung Disease) 2017 group C	Clinical Finding	Condition	SNOMED
36685454	GOLD (Global Initiative for Chronic Obstructive Lung Disease) 2017 group D	Clinical Finding	Condition	SNOMED

Markus AF, *et al. BMJ Open Respir Res* 2024; 11:e002127. doi: 10.1136/bmjresp-2023-002127

Concept ID	Name	Class	Domain ID	Vocabulary
36685455	GOLD (Global Initiative for Chronic Obstructive Lung Disease) 2017 spirometric grade 1	Clinical Finding	Condition	SNOMED
36685456	GOLD (Global Initiative for Chronic Obstructive Lung Disease) 2017 spirometric grade 2	Clinical Finding	Condition	SNOMED
36685457	GOLD (Global Initiative for Chronic Obstructive Lung Disease) 2017 spirometric grade 3	Clinical Finding	Condition	SNOMED
36685458	GOLD (Global Initiative for Chronic Obstructive Lung Disease) 2017 spirometric grade 4	Clinical Finding	Condition	SNOMED
4046986	End stage chronic obstructive airways disease	Clinical Finding	Condition	SNOMED
4110056	Chronic obstructive pulmonary disease with acute lower respiratory infection	Clinical Finding	Condition	SNOMED
4115044	Acute infective exacerbation of chronic obstructive airways disease	Clinical Finding	Condition	SNOMED
4193588	Moderate chronic obstructive pulmonary disease	Clinical Finding	Condition	SNOMED
4196712	Mild chronic obstructive pulmonary disease	Clinical Finding	Condition	SNOMED
4209097	Severe chronic obstructive pulmonary disease	Clinical Finding	Condition	SNOMED
44782563	Pulmonary hypertension due to chronic obstructive pulmonary disease	Clinical Finding	Condition	SNOMED
44788819	Mult COPD emerg hosp admission	Clinical Finding	Condition	SNOMED

Concept ID	Name	Class	Domain ID	Vocabulary
44791725	Very severe chronic obstructive pulmonary disease	Clinical Finding	Condition	SNOMED
44807895	Acute non-infective exacerbation of chronic obstructive pulmonary disease	Clinical Finding	Condition	SNOMED
46269701	Chronic obstructive lung disease co-occurrent with acute bronchitis	Clinical Finding	Condition	SNOMED

Online Supplement 3 – Respiratory drug classes

Initial Event Cohort:

People with continuous observation of 0 days before and 0 days after event may enter the cohort when observing any of the following:

- Drug exposure of drug class*.

Cohort Exit: If the index event is found within an era, the cohort end date will use the era's end date. Otherwise, it will use the observation period end date that contains the index event.

Table E6: Drug classes* are defined by the following ingredients and dose forms.

Drug class	ATC code	Ingredient	Concept ID	DDD	Included dose forms
ICS	R03BA01	beclomethasone	1115572	800 mcg	Inhalant, Inhalation Powder,
	R03BA02	budesonide	939259	800 mcg	Inhalation Solution,
	R03BA03	flunisolide	1196514	1000 mcg	Inhalation Spray, Inhalation
	R03BA05	fluticasone	1149380	600 mcg	Suspension, Metered Dose

Markus AF, *et al. BMJ Open Respir Res* 2024; 11:e002127. doi: 10.1136/bmjresp-2023-002127

	R03BA07	mometasone	905233	400 mcg	Inhaler, Dry Powder Inhaler,
	R03BA08	ciclesonide	902938	160 mcg	Gas for Inhalation
	R03BA04	betamethasone	920458	Not defined	
	R03BA06	triamcinolone	903963	Not defined	
	R03BA09	fluticasone furoate	Not available (falls under fluticasone)	Not defined	
SABA	R03AC02	salbutamol/albuterol	1154343	800 mcg	Inhalant, Inhalation Powder,
	R03AC03	terbutaline	1236744	2000 mcg	Inhalation Solution,
	R03AC04	fenoterol	19053979	600 mcg	Inhalation Spray, Inhalation
	R03AC05	rimiterol	19063387	1600 mcg	Suspension, Metered Dose
	R03AC06	hexoprenaline	19068969	1500 mcg	Inhaler, Dry Powder Inhaler,
	R03AC07	isoetharine	1181809	Not defined	Gas for Inhalation
	R03AC08	pirbuterol	1125449	1200 mcg	
	R03AC09	trimetoquinol hydrochloride hydrate	35198052	Not defined	
	R03AC10	carbuterol	40798689	Not defined	
	R03AC15	reproterol	19035396	Not defined	
	R03AC16	procaterol	19028950	60 mcg	
	R03AC17	bitolterol	1138050	Not defined	
LABA	R03AC11	tulobuterol	19043191	1600 mcg	Inhalant, Inhalation Powder,
	R03AC12	salmeterol	1137529	100 mcg	Inhalation Solution,
	R03AC13	formoterol	1196677	24 mcg	Inhalation Spray, Inhalation
	R03AC14	clenbuterol	19097824	Not defined	Suspension, Metered Dose
	R03AC18	indacaterol	40240664	150 mcg	Inhaler, Dry Powder Inhaler,
	R03AC19	olodaterol	45775116	5 mcg	Gas for Inhalation

Markus AF, *et al. BMJ Open Respir Res* 2024; 11:e002127. doi: 10.1136/bmjresp-2023-002127

	Not available	vilanterol	43532539	Not defined	
SAMA	R03BB01	ipratropium	1112921	120 mcg	Inhalant, Inhalation Powder, Inhalation Solution, Inhalation Spray, Inhalation Suspension, Metered Dose Inhaler, Dry Powder Inhaler, Gas for Inhalation
	R03BB02	oxitropium	19018882	600 mcg for inhalation aerosol, 4000 mcg for inhalation solution	
LAMA	R03BB04	tiotropium	1106776	10 mcg (DPI) 5 mcg (soft mist inhaler)	Inhalant, Inhalation Powder, Inhalation Solution, Inhalation Spray, Inhalation Suspension, Metered Dose Inhaler, Dry Powder Inhaler, Gas for Inhalation
	R03BB05	aclidinium	42873639	644 mcg	
	R03BB06	glycopyrronium	45775571	44 mcg	
	R03BB07	umeclidinium	44785907	55 mcg	
LTRA	R03DC01	zafirlukast	1111706	40 mg	Buccal Tablet, Delayed Release Oral Tablet, Disintegrating Oral Tablet, Chewable Tablet, Delayed Release Oral Capsule, Extended Release Oral Tablet, Extended Release Suspension, Oral Capsule, Oral Gel, Oral Granules, Oral Lozenge, Oral Ointment, Oral
	R03DC02	pranlukast hydrate	43009065	Not defined	
	R03DC03	montelukast	1154161	10 mg	
	R03DC04	ibudilast	43009091	Not defined	

					Paste, Oral Powder, Oral Solution, Oral Suspension, Effervescent Oral Tablet, Extended Release Oral Capsule, Powder for Oral Suspension, Sublingual Tablet, Tablet for Oral Suspension, Oral Tablet, Pack
Xanthines	R03DA01	diprophylline/dyphylline	1140088	1 g	Buccal Tablet, Delayed Release Oral Tablet, Disintegrating Oral Tablet, Chewable Tablet, Delayed Release Oral Capsule, Extended Release Oral Tablet, Extended Release Suspension, Oral Capsule, Oral Gel, Oral Granules, Oral Lozenge, Oral Ointment, Oral Paste, Oral Powder, Oral Solution, Oral Suspension, Effervescent Oral Tablet, Extended Release Oral Capsule, Powder for Oral Suspension, Sublingual Tablet, Tablet for Oral Suspension, Oral Tablet, Pack
	R03DA02	choline theophyllinate	1195334	600 mg	
	R03DA03	proxyphylline	19029547	1.2 g	
	R03DA04	theophylline	1237049	400 mg	
	R03DA05	aminophylline	1105775	600 mg	
	R03DA06	etamiphyllin	40798802	Not defined	
	R03DA07	theobromine	19137056	Not defined	
	R03DA08	bamifylline	19018518	Not defined	
	R03DA09	acefylline piperazine	40798596	Not defined	
	R03DA10	bufylline	Not available	Not defined	
	R03DA11	doxofylline	43009019	800 mg	

Systemic glucocorticosteroids	H02AB01	betamethasone	920458	1.5 mg	Injectable Solution, Injectable Suspension, Auto-Injector, Intravenous Solution, Prefilled Syringe, Injection, Intramuscular Solution, Buccal Tablet, Delayed Release Oral Tablet, Disintegrating Oral Tablet, Chewable Tablet, Delayed Release Oral Capsule, Extended Release Oral Tablet, Extended Release Suspension, Oral Capsule, Oral Gel, Oral Granules, Oral Lozenge, Oral Ointment, Oral Paste, Oral Powder, Oral Solution, Oral Suspension, Effervescent Oral Tablet, Extended Release Oral Capsule, Powder for Oral Suspension, Sublingual Tablet, Tablet for Oral Suspension, Oral Tablet, Pack
	H02AB02	dexamethasone	1518254	1.5 mg	
	H02AB03	fluocortolone	19055344	10 mg	
	H02AB04	methylprednisolone	1506270	7.5 mg	
	H02AB05	paramethasone	19027186	4 mg	
	H02AB06	prednisolone	1550557	10 mg	
	H02AB07	prednisone	1551099	10 mg	
	H02AB08	triamcinolone	903963	7.5 mg	
	H02AB09	hydrocortisone	975125	30 mg	
	H02AB10	cortisone	1507705	37.5 mg	
	H02AB11	prednylidene	19011127	12 mg	
	H02AB12	rimexolone	977421	Not defined	
	H02AB13	deflazacort	19086888	15 mg	
	H02AB14	cloprednol	19050907	Not defined	
	H02AB15	meprednisone	19009116	Not defined	
	H02AB17	cortivazol	19061907	Not defined	
	H02AB90	flumetasone	19055156	Not defined	
Anti-IgE	R03DX05	omalizumab	1110942	16 mg	Injectable Solution, Injectable Suspension, Auto-Injector, Intravenous Solution, Prefilled Syringe,

					Injection, Intramuscular Solution
Anti-IL4Rα	D11AH05	dupilumab	1593467	21.4 mg	Injectable Solution, Injectable Suspension, Auto-Injector, Intravenous Solution, Prefilled Syringe, Injection, Intramuscular Solution
Anti-IL5(R)	R03DX08	reslizumab	35603983	7.1 mg	Injectable Solution, Injectable Suspension, Auto-Injector, Intravenous Solution, Prefilled Syringe, Injection, Intramuscular Solution
	R03DX09	mepolizumab	35606631	3.6 mg	
	R03DX10	benralizumab	792993	0.54 mg	
PDE4	R03DX07	roflumilast	40236897	500 mcg	Buccal Tablet, Delayed Release Oral Tablet, Disintegrating Oral Tablet, Chewable Tablet, Delayed Release Oral Capsule, Extended Release Oral Tablet, Extended Release Suspension, Oral Capsule, Oral Gel, Oral Granules, Oral Lozenge, Oral Ointment, Oral Paste, Oral Powder, Oral Solution, Oral Suspension, Effervescent Oral Tablet, Extended Release Oral

		Capsule, Powder for Oral Suspension, Sublingual Tablet, Tablet for Oral Suspension, Oral Tablet, Pack

Online Supplement 4 – Baseline characteristics

Table E7: Code list of baseline characteristics.

Name	Concept IDs (any time prior to first diagnosis)
Sex (Male)	x
Age (in years)	x
Charlson comorbidity index score	x
Anxiety	441542
Allergic rhinitis	257007
Atopic disorders	133834
Cerebrovascular disease (stroke and/or TIA)	381591
Chronic rhinosinusitis (allergic fungal sinusitis, chronic rhinitis, chronic sinusitis)	132932,134661,134668,139841,257012,259848,761761,761762, 765276,4048184,4048185,4051475,4051486,4051487,4051488, 4110027,4110489,4110490,4112365,4112367,4112497,4112498, 4112529,4145495,4173466,4179673,4181738,4230641,4247588, 4288156,4316066,4316067,4322228
Depressive disorder	440383
Diabetes mellitus (type I and II)	201820
Gastroesophageal reflux disease	318800,765110,4046097,4076267,4144111,4159148,4159156, 36687117,36712768,36712969,36713492,36713493,42535063
Heart failure	316139
Hypertensive disorder	316866
Ischemic heart disease (angina pectoris and/or myocardial infarction)	4185932
Lower Respiratory Tract Infection (previous year)	4028876
Nasal polyposis	42537251
Obesity	433736

Details of Concept IDs can be found on https://athena.ohdsi.org/.

Markus AF, et al. BMJ Open Respir Res 2024; 11:e002127. doi: 10.1136/bmjresp-2023-002127

Online Supplement 5 – Study settings treatment trajectories

Table E8: Study settings treatment trajectories.

**General settings**		
studyName	Unique name identifying the set of study parameters below	Main analysis
targetCohortId	Select one study population	Asthma or COPD
eventCohortIds	Select all treatments of interest	All respiratory drug classes[1]
**Analysis settings**		
includeTreatmentsPriorToIndex	Number of days prior to the index date of the target cohort that event cohorts are allowed to start	0[2]
minEraDuration	Minimum time an event era should last to be included in analysis	5[3]
splitEventCohorts	Specify event cohort to split in acute (< 30 days) and therapy (≥ 30 days)	Systemic glucocorticoids
eraCollapseSize	Window of time between which two eras of the same event cohort are collapsed into one era	30
combinationWindow	Window of time two event cohorts need to overlap to be considered a combination treatment	30
minStepDuration	Minimum time an event era before or after a generated combination treatment should last to be included in analysis	30
filterTreatments	Select first occurrences of / changes between / all event cohorts	Changes
**Output settings**		
maxPathLength	Maximum number of steps included in treatment trajectory	5
minCellCount	Minimum number of persons with a specific treatment trajectory for the trajectory to be included in analysis	5
minCellMethod	Select to completely remove / sequentially adjust (by removing last step as often as necessary) treatment trajectories below minCellCount	Adjust
groupCombinations	Select to group all non-fixed combinations in one category 'other' in the sunburst plot	10
addNoPaths	Select to include untreated persons without treatment trajectory in the sunburst plot	TRUE

[1] Sensitivity analysis 1) restricting to inhaled medications only
[2] Sensitivity analysis 2) including respiratory drugs initiated up to 30 days before the first diagnosis
[3] Sensitivity analysis 3) lifting the criterium that drug eras should last at least 5 days

Markus AF, *et al. BMJ Open Respir Res* 2024; 11:e002127. doi: 10.1136/bmjresp-2023-002127

Online Supplement 6 – Step-wise treatment

We labelled switches between treatments with two definitions:

1. *A strict definition* following clinical guideline recommendations. Categorizing switches in treatments conform the list of possibilities below (if a switch is not included it is marked as 'Not according to guidelines'). This results in a clean definition, but leads to undefined switches due to heterogeneity of observational data.

Table E9: Definition following GINA guidelines [1] – Asthma.

From	To	Label
SABA	SABA+SAMA	step up
SABA	ICS+SABA	step up
SABA	ICS	step up
SABA	LTRA	step up
SAMA	SABA+SAMA	step up
SAMA	ICS+SABA	step up
SAMA	ICS	step up
SAMA	LTRA	step up
ICS	ICS+LABA	step up
ICS	ICS+LAMA	step up
ICS	ICS+LTRA	step up
ICS+LABA	ICS+LABA+LAMA	step up
ICS+LABA	ICS+LABA+Systemic glucocorticoids (therapy)	step up
ICS+LABA	Anti-IgE+ICS+LABA	step up
ICS+LABA	Anti-IL5(R)+ICS+LABA	step up
ICS+LABA	Anti-IL4Rα+ICS+LABA	step up

ICS+LTRA	ICS+LABA+LAMA	step up
ICS+LTRA	ICS+LABA+LAMA	step up
ICS+LTRA	ICS+LABA+Systemic glucocorticoids (therapy)	step up
ICS+LTRA	Anti-IgE+ICS+LABA	step up
ICS+LTRA	Anti-IL5(R)+ICS+LABA	step up
ICS+LTRA	Anti-IL4Rα+ICS+LABA	step up
SABA	SAMA	switching
SAMA	SABA	switching
ICS	LTRA	switching
LTRA	ICS	switching
ICS+LABA	ICS+LTRA	switching
ICS+LTRA	ICS+LABA	switching
ICS+LABA	ICS+LAMA	switching
ICS+LAMA	ICS+LABA	switching
ICS+LABA+LAMA	ICS+LABA+Systemic glucocorticoids (therapy)	switching
ICS+LABA+LAMA	Anti-IgE+ICS+LABA	switching
ICS+LABA+LAMA	Anti-IL5(R)+ICS+LABA	switching
ICS+LABA+LAMA	Anti-IL4Rα+ICS+LABA	switching
ICS+LABA+Systemic glucocorticoids (therapy)	ICS+LABA+LAMA	switching
ICS+LABA+Systemic glucocorticoids (therapy)	Anti-IgE+ICS+LABA	switching
ICS+LABA+Systemic glucocorticoids (therapy)	Anti-IL5(R)+ICS+LABA	switching
ICS+LABA+Systemic glucocorticoids (therapy)	Anti-IL4Rα+ICS+LABA	switching

Anti-IgE+ICS+LABA	ICS+LABA+Systemic glucocorticoids (therapy)	switching
Anti-IgE+ICS+LABA	Anti-IL5(R)+ICS+LABA	switching
Anti-IgE+ICS+LABA	Anti-IL4Rα+ICS+LABA	switching
Anti-IL5(R)+ICS+LABA	ICS+LABA+LAMA	switching
Anti-IL5(R)+ICS+LABA	Anti-IgE+ICS+LABA	switching
Anti-IL5(R)+ICS+LABA	Anti-IL4Rα+ICS+LABA	switching
Anti-IL4Rα+ICS+LABA	ICS+LABA+Systemic glucocorticoids (therapy)	switching
Anti-IL4Rα+ICS+LABA	ICS+LABA+LAMA	switching
Anti-IL4Rα+ICS+LABA	Anti-IgE+ICS+LABA	switching
Anti-IL4Rα+ICS+LABA	Anti-IL5(R)+ICS+LABA	switching
SABA+SAMA	SABA	step down
ICS+SABA	SABA	step down
ICS	SABA	step down
LTRA	SABA	step down
SABA+SAMA	SAMA	step down
ICS+SABA	SAMA	step down
ICS	SAMA	step down
LTRA	SAMA	step down
ICS+LABA	ICS	step down
ICS+LAMA	ICS	step down
ICS+LTRA	ICS	step down
ICS+LABA+LAMA	ICS+LABA	step down
ICS+LABA+Systemic glucocorticoids (therapy)	ICS+LABA	step down
Anti-IgE+ICS+LABA	ICS+LABA	step down
Anti-IL5(R)+ICS+LABA	ICS+LABA	step down

Anti-IL4Rα+ICS+LABA	ICS+LABA	step down
ICS+LABA+LAMA	ICS+LTRA	step down
ICS+LABA+LAMA	ICS+LTRA	step down
ICS+LABA+Systemic glucocorticoids (therapy)	ICS+LTRA	step down
Anti-IgE+ICS+LABA	ICS+LTRA	step down
Anti-IL5(R)+ICS+LABA	ICS+LTRA	step down
Anti-IL4Rα+ICS+LABA	ICS+LTRA	step down

Table E10: Definition following GOLD guidelines [2] – COPD.

From	To	Label
SABA	SABA+SAMA	step up
SABA	LABA	step up
SABA	LAMA	step up
SABA	Xanthines	step up
SAMA	SABA+SAMA	step up
SAMA	LABA	step up
SAMA	LAMA	step up
SAMA	Xanthines	step up
LABA	LABA+LAMA	step up
LABA	ICS+LABA	step up
LABA	ICS+LAMA	step up
LAMA	LABA+LAMA	step up
LAMA	ICS+LABA	step up
LAMA	ICS+LAMA	step up
Xanthines	LABA+LAMA	step up
Xanthines	ICS+LABA	step up
Xanthines	ICS+LAMA	step up

LABA+LAMA	ICS+LABA+LAMA	step up
ICS+LABA	ICS+LABA+LAMA	step up
ICS+LABA	ICS+LABA+PDE4	step up
ICS+LAMA	ICS+LABA+LAMA	step up
SABA	SAMA	switching
SAMA	SABA	switching
LABA	LAMA	switching
LABA	Xanthines	switching
LAMA	LABA	switching
LAMA	Xanthines	switching
Xanthines	LABA	switching
Xanthines	LAMA	switching
LABA+LAMA	ICS+LABA	switching
LABA+LAMA	ICS+LAMA	switching
ICS+LABA	LABA+LAMA	switching
ICS+LABA	ICS+LAMA	switching
ICS+LAMA	LABA+LAMA	switching
ICS+LAMA	ICS+LABA	switching
ICS+LABA+LAMA	ICS+LABA+PDE4	switching
ICS+LABA+PDE4	ICS+LABA+LAMA	switching
SABA+SAMA	SABA	step down
LABA	SABA	step down
LAMA	SABA	step down
Xanthines	SABA	step down
SABA+SAMA	SAMA	step down
LABA	SAMA	step down
LAMA	SAMA	step down
Xanthines	SAMA	step down

LABA+LAMA	LABA	step down
ICS+LABA	LABA	step down
ICS+LAMA	LABA	step down
LABA+LAMA	LAMA	step down
ICS+LABA	LAMA	step down
ICS+LAMA	LAMA	step down
LABA+LAMA	Xanthines	step down
ICS+LABA	Xanthines	step down
ICS+LAMA	Xanthines	step down
ICS+LABA+LAMA	LABA+LAMA	step down
ICS+LABA+LAMA	ICS+LABA	step down
ICS+LABA+PDE4	ICS+LABA	step down
ICS+LABA+LAMA	ICS+LAMA	step down

2. A *broad definition* capturing the clinical interpretation of the guidelines. This definition categorizes all possible switches by defining drug class levels and subsequent patient treatment levels (sum of drug class levels received by a patient), see explanation below.

We distinguish the following labels:

- Step up
- Step down
- Switching
- Start of acute exacerbation
- End of acute exacerbation
- No follow-up treatment with a different drug class
- Other (= possibly off-label use)†

To categorize the switches each treatment will first receive a score that determines the *patient treatment level*. The patient treatment level is computed by summing the *drug class levels* corresponding to each received treatment at a given point in time (e.g. 'ICS + LABA' = 1 + 2 = Patient treatment level 3). The drug class levels were determined by clinical experts and can be found in Table E11. A higher drug class level indicates medications that are seen as more intensive treatment (relative to others). Table E12 contains examples of resulting patient treatment levels for common asthma treatments.

Table E11: Drug class levels for asthma and COPD.

	**For asthma**	**For COPD**
**Level 0**	SABA	SABA
**(relievers)**	SAMA	SAMA
	Systemic ß2 agonist	Systemic ß2 agonist
**Level 1**	ICS	
	LTRA	LABA
	PDE4[†]	LAMA
	Xanthines	Xanthines
**Level 2**		ICS
	LABA[†]	LTRA[†]
	LAMA[†]	PDE4
**Level 3**	Systemic glucocorticosteroids (therapy)	Systemic glucocorticosteroids (therapy)
		Biologic:
	Biologic:	Anti-IL5(R)[†]
	Anti-IL5(R)	Anti-IgE[†]

Anti-IgE	Anti-IL4Rα[†]
Anti-IL4Rα	

Table E12: Examples of resulting patient treatment levels for asthma.

**Level 0**	SABA, SAMA, SABA + SAMA
**Level 1**	ICS, LTRA, ICS+SABA,
**Level 2**	ICS+LTRA
**Level 3**	ICS+LABA, ICS+LAMA, Systemic glucocorticoids
**Level 4**	LABA + LAMA, ICS+LABA+LTRA, ICS+LAMA+LTRA, ICS+Systemic glucocorticosteroids, LTRA+ Systemic glucocorticosteroids
**Level 5**	ICS+LABA+LAMA
**Level 6+**	ICS+LABA+LAMA+LTRA (6),<br>ICS+LABA+LAMA+Systemic glucocorticosteroids (8),<br>ICS+LABA+LAMA+Biologic (8)<br>…

* The sum of the individual drug class levels received determines patient treatment level (capped at level 6).

Finally, labels will be assigned based on the change in patient treatment level. For example, a change from 'SABA + SAMA' (Patient treatment level 0) to 'ICS + LABA' (Patient treatment level 3) indicates a change to a higher patient treatment level and translates to 'Step up'. The following rules are applied to assign labels (in this order, with later rules overruling the earlier):

- To higher patient treatment level -> 'Step up'
- To lower patient treatment level -> 'Step down'
- To same patient treatment level -> 'Switching'

- To 'Systemic glucocorticoids (acute)' -> 'Start of acute exacerbation'
- From 'Systemic glucocorticoids (acute)' -> 'End of acute exacerbation'
- To PDE4, LABA (without ICS), LAMA (without ICS) for asthma -> 'Other'
- To LTRA, Anti-IL5(R), Anti-IgE, Anti-IL4Rα for COPD -> 'Other'
- Else -> 'No follow-up treatment with different drug class'

Online Supplement 7 – Results treatment step-up/step-down

The sensitivity analyses using the strict definition of treatment step-up/step-down showed similar patterns for adults with asthma (Table E13) and COPD (Table E14).

Table E13: Percentage of adults with asthma who switched, stepped-down or stepped-up respiratory pharmacological treatment after the first-line treatment (strict definition).

Label	CCAE (United States)	Medicaid (United States)	Medicare (United States)	EHIF (Estonia)	CPRD (United Kingdom)	SIDIAP (Spain)	IPCI (Netherlands)	AUSOM (South Korea)
Not according to guidelines	58.5	54.0	56.5	45.6	52.1	62.1	46.6	67.6
Step-up	5.5	5.4	4.4	10.4	15.6	8.4	8.9	0.9
Step-down	3.5	2.9	2.6	13.1	12.7	7.6	5.3	0.09
Switching	0.4	0.3	0.4	1.0	0.4	1.4	0.7	0.8
No follow-up treatment*	32.0	37.5	36.0	29.9	19.1	20.6	38.4	30.6

* Patients who did not receive medication of a different respiratory drug class after the first treatment, i.e. patients who remained on the same treatment or who discontinued treatment.

Markus AF, et al. BMJ Open Respir Res 2024; 11:e002127. doi: 10.1136/bmjresp-2023-002127

Table E14: Percentage of adults with COPD who switched, stepped-down or stepped-up respiratory pharmacological treatment after the first-line treatment (strict definition).

**Label**	**CCAE (United States)**	**Medicaid (United States)**	**Medicare (United States)**	**EHIF (Estonia)**	**CPRD (United Kingdom)**	**SIDIAP (Spain)**	**IPCI (Netherlands)**	**AUSOM (South Korea)**
Not according to guidelines	57.2	57.5	54.1	43.5	71.4	65.2	56.4	47.3
Step-up	1.9	3.3	3.0	8.4	10.9	8.0	10.6	1.1
Step-down	1.7	2.3	2.2	5.4	4.9	4.5	5.2	2.6
Switching	0.3	0.3	0.3	3.6	1.3	2.9	2.2	6.0
No follow-up treatment*	39.0	36.5	40.4	39.0	11.6	19.3	25.6	43.0

* Patients who did not receive medication of a different respiratory drug class after the first treatment, i.e. patients who remained on the same treatment or who discontinued treatment.

Abbreviations: CCAE = IBM MarketScan® Commercial Claims and Encounters Database, Medicaid = IBM MarketScan® Multi-State Medicaid Database, Medicare = IBM MarketScan® Medicare Supplemental Database, EHIF = Estonian Health Insurance Fund, CPRD = Clinical Practice Research Datalink, SIDIAP = Information System for Research in Primary Care, IPCI = Integrated Primary Care Information, AUSOM = Ajou University School of Medicine.

Online Supplement 8 – Sensitivity analysis

Since we could not ensure that the moment of diagnosis of asthma or COPD was similar across all databases, care settings, and countries, as this is likely to be influenced by a variety of factors inherent to the different health care systems, we performed three sensitivity analyses for each study population: 1) restricting to inhaled medications only, 2) including respiratory drugs initiated up to 30 days before the first diagnosis, and 3) lifting the criterium that drug eras should last at least 5 days. Results can be explored in can be explored in the interactive online Shiny application: https://mi-erasmusmc.shinyapps.io/AsthmaCOPDTreatmentPatterns/. Importantly, these had no significant impact on the observed treatment trajectories.

Furthermore, since differentiating asthma from COPD might be challenging (especially in primary care and at first presentation), we also investigated treatment trajectories in newly diagnosed patients with asthma COPD overlap (ACO).

Study population:

iii) Asthma-COPD overlap

Initial Event Cohort:

People with continuous observation of 365 days before and 1,095 days after event may enter the cohort when observing any of the following:

- Condition occurrence of COPD** for the first time in the person's history
    - with at least one condition occurrence of Asthma* all days before and 0 days after index start date.

- Condition occurrence of Asthma* for the first time in the person's history
    - with at least one condition occurrence of COPD** all days before and 0 days after index start date.

Inclusion Criteria:

- Age >= 40 years AND,
- Index date after December 31, 2009 and before December 31, 2019.

Cohort Exit: the person exits the cohort at the end of continuous observation.

For code lists see Online Supplement 2 for asthma (Table E4) and COPD (Table E5).

Results:

Table E15: Baseline characteristics of adults with ACO.

Characteristic	CCAE (United States)	Medicaid (United States)	Medicare (United States)	EHIF (Estonia)	CPRD (United Kingdom)	SIDIAP (Spain)	IPCI (Nether lands)	AUSOM (South Korea)
Number of patients	84,171	128,664	67,785	13,182	16,606	10,637	2,410	552
Sex: Male, %	37.4	28.4	41.4	46	45.2	49.8	45.5	71.7
Age at index (years), Mean	53.9	58.6	75.1	66.5	64.3	66.5	63.3	65.8
Charlson Comorbidity Index, Mean	2.6	4.1	4.3	2.7	2	2	1.8	2.2
Common comorbid conditions, % (any time prior first diagnosis)								
- Anxiety	26.2	47.9	15.7	8.3	29.8	27	22.7	6.2
- Atopic disorders	3.4	2.3	3.2	2.3	12.2	2.6	13.1	2.2
- Allergic rhinitis	38.2	29.9	26.8	8.9	13.2	12.6	18.1	16.3
- Cerebrovascular disease	10.8	19.2	34.9	8.5	6.3	3.1	7.7	4.2
- Chronic rhinosinusitis	29.4	20.7	21.6	6.4	9.3	1.9	15.2	12.7
- Depressive disorder	28	53.6	18.6	12.5	35.4	18.1	20.8	5.4
- Diabetes mellitus	27.5	45.3	37.6	16.1	12.6	19	17.1	15.4
- Gastroesophageal reflux disease	35.9	54.2	36.6	13.4	6	6.6	4.7	12.5
- Heart failure	8.4	24.5	27	42	3.1	6.8	6.7	5.4
- Hypertensive disorder	62.1	82.4	84.7	36.2	38	53.1	37.9	44.2
- Ischemic heart disease	13.9	22.9	30.9	25.4	14	8.4	14.4	14
- Nasal polyposis	2.2	0.6	1.6	1.6	3	2.7	0.8	3.4
- Obesity	23.2	36.4	14	15.4	7.4	30.8	10.2	1.1
- Lower respiratory tract infections (previous year)	45.9	49.4	50.7	38.6	6.1	25.2	21.7	21.4

Markus AF, *et al. BMJ Open Respir Res* 2024; 11:e002127. doi: 10.1136/bmjresp-2023-002127

Table E16: Number of patients per year with ACO.

Characteristic	CCAE (United States)	Medicaid (United States)	Medicare (United States)	EHIF (Estonia)	CPRD (United Kingdom)	SIDIAP (Spain)	IPCI (Nether lands)	AUSOM (South Korea)
Year 2010	11720	12117	12259		2959	1018	261	63
Year 2011	12225	11217	10794		2875	913	263	74
Year 2012	9648	10577	10843	3404	2515	1211	324	55
Year 2013	9556	15748	10410	2704	2166	1502	387	89
Year 2014	11441	14015	10041	2499	1886	1461	348	59
Year 2015	10639	21757	5145	2403	1714	1414	300	44
Year 2016	8717	23581	4194	2172	1742	1199	345	84
Year 2017	7727	19611	3016		749	1114	182	55
Year 2018+	2498	41	1083			805		29
**Total**	**84,171**	**128,664**	**67,785**	**13,182**	**16,606**	**10,637**	**2,410**	**552**

Figure E1: Sunburst plots of adults with ACO showing the first respiratory pharmacological treatment in the center and subsequent pharmacological treatments in the surrounding outer layers. Each color represents a respiratory drug class. A layer with multiple colors indicates a loose combination therapy. The number of patients (N) and percentage of patients treated are indicated for each database.

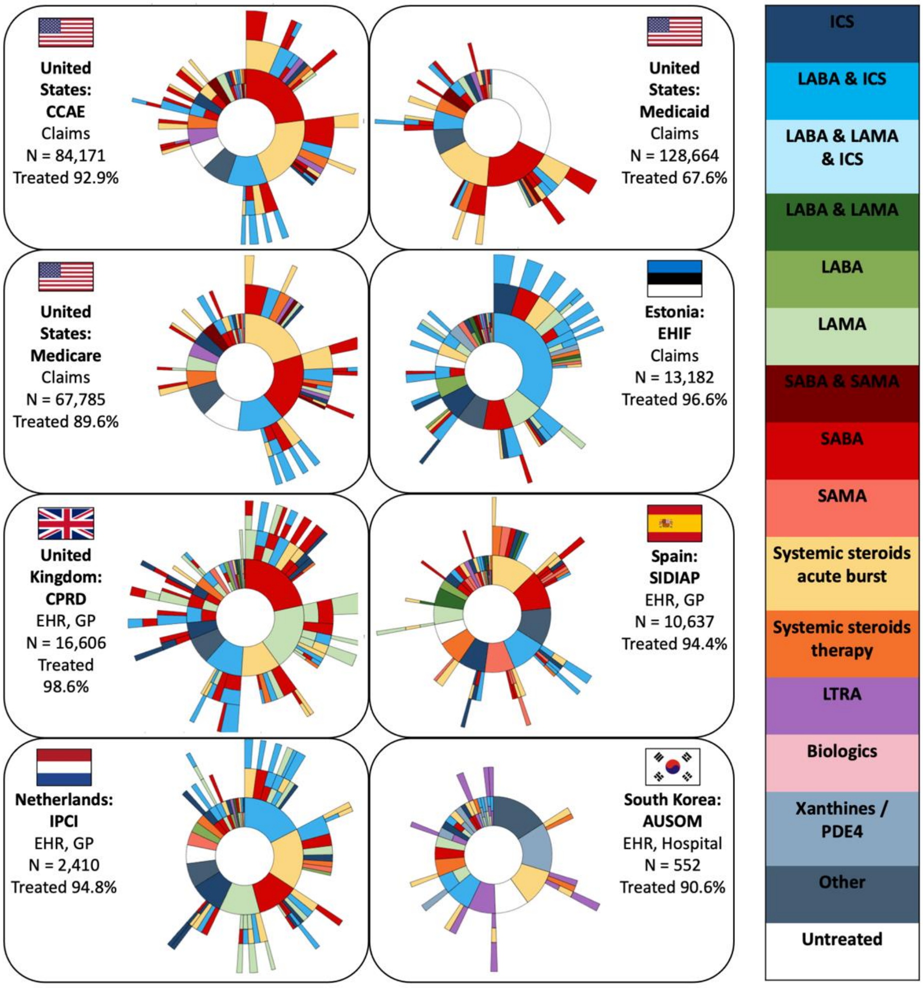

Online Supplement 9 – Pooled results

Figure E2: Pooled sunburst plots of adults with asthma, COPD, and ACO across databases (left column: Claims United States including CCAE, Medicaid, Medicare and right column: EHR Europe including CPRD, SIDIAP, IPCI).

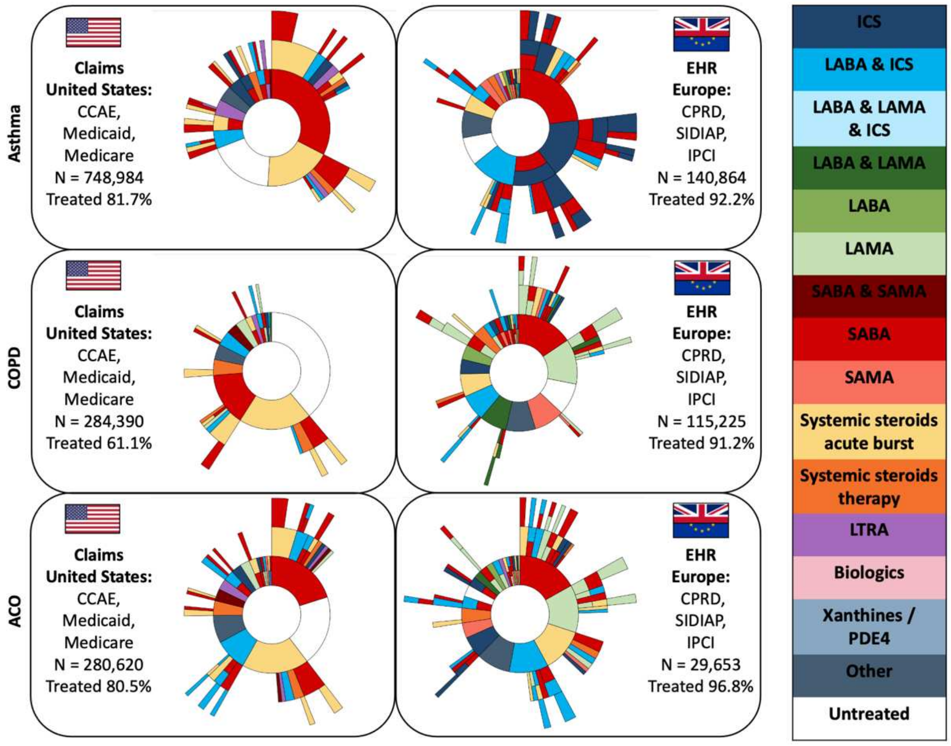

Markus AF, *et al. BMJ Open Respir Res* 2024; 11:e002127. doi: 10.1136/bmjresp-2023-002127

References

1. Global Initiative for Asthma. Global strategy for asthma management and prevention. 2021 [last accessed 2021 June]; Available from: https://ginasthma.org/wp-content/uploads/2021/05/GINA-Main-Report-2021-V2-WMS.pdf

2. Global Initiative for Chronic Obstructive Lung Disease. Global strategy for prevention, diagnosis and management of copd. 2021 [last accessed 2021 June]; Available from: https://goldcopd.org/wp-content/uploads/2020/11/GOLD-REPORT-2021-v1.1-25Nov20_WMV.pdf
